# Impact of heartfulness meditation on psychophysiological parameters among medical students

**DOI:** 10.6026/973206300214643

**Published:** 2025-12-15

**Authors:** Anupama Gupta, Suniti Pandey, Rajeshwari Hegde, Tambe J.J

**Affiliations:** 1 Department of Physiology, G.S.V.M. Medical College, Kanpur, Uttar Pradesh, India; 2 Department of Anatomy, G.S.V.M. Medical College, Kanpur, Uttar Pradesh, India; 3 Department of Electronics and Telecommunication, B.M.S. College of Engineering, Bangalore, Karnataka, India; 4 Pediatrician, C.G.H.S. Hospital, Kanpur, Uttar Pradesh, India

**Keywords:** Indian National Medical Commission (NMC), Competency-Based Medical Education (CBME), heartfulness meditation, yogic transmission

## Abstract

The shift from school to medical college is a demanding phase, during which many first-year MBBS students encounter stress, emotional strain and physiological imbalance that can hinder academic performance and personal well-being. In response, the Indian National Medical Commission has integrated yoga and meditation practices into the Foundation Course under the Competency-Based Medical Education framework to promote resilience and holistic growth. Hence, a cross-sectional observational study was conducted at GSVM Medical College, Kanpur, across five student cohorts, where participants engaged in a single 20-25-minute Heartfulness meditation session with yogic transmission. Following the intervention, nearly 90% of students experienced deep relaxation and statistically significant reductions (p < 0.001) in pulse and respiratory rates were observed, indicating immediate physiological and psychological benefits.

## Background:

The transition from pre-university education to a professional medical curriculum is widely recognized as one of the most stressful phases in a student's academic journey. The initial phase of medical education often subjects Phase-1 MBBS students to considerable psychological stress, emotional strain and physiological imbalances [1]. Adapting to the rigorous academic environment and high expectations can impair key cognitive processes including memory, attention and emotional regulation, potentially contributing to early burnout or detachment from the profession [2]. To address these challenges, the National Medical Commission (NMC) of India has integrated Yoga and wellness modules into the Foundation Course under the Competency-Based Medical Education (CBME) framework. This curriculum aims to foster holistic development, enhance self-awareness and promote resilience to stress among future healthcare professionals [3]. While yoga broadly encompasses physical postures, breathing techniques and spiritual practices, meditation-acore element of the Ashtanga Yoga-has gained scientific recognition for its role in emotional regulation and autonomic stability [4]. One such approach is Heartfulness Meditation (HM), rooted in the Raja Yoga tradition. This heart-based, secular and easily scalable method emphasizes inner stillness and balance, supported by a distinctive feature known as Yogic Transmission (Pranahuti), which is believed to deepen the meditative experience with minimal effort or prior training [5]. Practicing Heartfulness meditation is associated with calming parasympathetic activity through vagal stimulation, contributing to reductions in pulse and respiratory rates and fostering a state of emotional calm, clarity and expanded awareness [6, 7 and 8]. Despite its growing global relevance in educational and healthcare settings, empirical evidence on its immediate physiological and psychological effects on medical students is limited, especially in populations with no prior exposure to meditation. Moreover, research exploring consciousness-related changes, such as decreased thought activity, altered body awareness, or feelings of lightness and peace, is still in its early stages [9, 10]. The present study was conducted at a tertiary care medical institute in Kanpur and included five consecutive cohorts of newly admitted MBBS students during the Foundation Course. As part of the orientation program, each cohort underwent a single session of guided His artfulness Meditation. Data was collected over a period of five academic years (2021-2024), with a total of 1,079 participants, representing one of the largest initiatives of its kind reported from India Related Work. Tang *et al.* reported that short-term integrative body-mind training significantly decreases activity of sympathetic nervous system and enhanced vagal tone in young adults [11]. Similarly, Zeidan *et al.* demonstrated that as few as four brief sessions of mindfulness meditation can significantly enhance attentional control, diminish mind-wandering and improve working memory-effects that closely parallel the reduction in cognitive clutter and the enhancement of mental clarity reported by participants in our study [10]. Telles *et al.* conducted research and found that meditative breathing practices based on Yoga improved parasympathetic activation and subjective well-being among students [12]. Similar results were found from other meditative practices also. It was found that Transcendental Meditation reduced blood pressure and improved autonomic balance through enhanced parasympathetic activity [13]. Kriya, a structured, rhythmic Yoga technique has also been shown to reduce stress-related biomarkers such as cortisol and improve vagal tone indicators. The contemplative practices share a common physiological mechanism though the techniques used are different [19, 20- 21]. As a result, the sympathetic nervous system activity is reduced and the parasympathetic nervous system activity is increased, resulting in improved emotional regulation and physical well-being [14]. According to yogic philosophy, consciousness can be understood as a spectrum ranging from deep unconsciousness to heightened self-awareness. Drawing from both yogic philosophy and neuroscience, this continuum is mapped using the symbolic sounds of AUM- 'A' representing the alert waking state, 'U' denoting the dreamlike state and 'M' corresponding to deep sleep or Sushupti. Beyond these, the silent state following 'M', known as Turiya, reflects a transcendental level of consciousness in which one is deeply at peace yet fully aware. Remarkably, this state is accessible not only to experienced meditators but also to beginners when supported by Yogic Transmission in Heartfulness Meditation [4]. In this condition, the body remains profoundly relaxed while the mind remains conscious, offering a sense of inner expansion akin to deep rest. With continued practice, this state can be carried into daily life, evolving into the Turiyatit condition-where meditative awareness becomes constant. Such expanded states of consciousness not only foster inner growth but also enhance leadership abilities, decision-making and emotional balance, making them highly relevant in both spiritual and professional domains [4]. This experience aligns with the polyvagal theory, which emphasizes the role of the ventral vagal complex in fostering emotional balance, adaptive stress responses and social engagement [15, 8]. Emerging neuroscientific evidence further supports the idea that meditative practices like Heartfulness can promote neuroplasticity, improve heart-brain coherence and strengthen interpersonal connectedness [16, 17-18]. These findings demonstrate how ancient contemplative frameworks intersect with contemporary neuroscience and psychology, offering a comprehensive model for enhancing human well-being [4, 9]. Therefore, it is of interest to evaluate the Impact of heartfulness meditation on physiological and psychological parameters among medical students: a multi-cohort observational study.

## Materials and Methods:

## Study design and setting:

This study was a multi-cohort, observational, cross-sectional study, conducted annually over five academic years (2021-2022 to 2024-2025 batches) at Ganesh Shankar Vidyarthi Medical College, Kanpur, a government-run tertiary medical teaching institution in North India. Each academic batch of newly admitted Phase I MBBS students constituted an independent cohort; no longitudinal follow-up was performed. The intervention was integrated into the Foundation Course, as mandated by the National Medical Commission (NMC), specifically under the stress management and wellness module. As a component of this module, students were introduced to Heartfulness Meditation a heart-based meditation technique rooted in Raja Yoga. The practice emphasizes pratyahara (withdrawal of senses) and dhyana (meditative absorption), core limbs of Ashtanga Yoga. The meditation sessions were conducted in a designated lecture theatre, which was maintained in a quiet, temperature-controlled and distraction-free environment, thereby enhancing the potential for inward focus and meditative depth.

## Ethical Considerations:

The study received approval from the Institutional Ethics Committee (IEC), GSVM Medical College, Kanpur. Written informed consent was obtained from all participants after being briefed about the study objectives and procedures. Anonymity and confidentiality of participant data were ensured throughout the research.

## Participants:

A total of 1,079 Phase 1 MBBS students participated over five consecutive academic years. Participants ranged in age from 18 to 25 years and had no prior exposure to formal meditation techniques. The batch-wise distribution was as follows: n = 245, 190, 209, 209, 226 (for batches 2020- 21 to 2024-25, respectively). The inclusion and exclusion criteria are given below. The inclusion and exclusion criteria to participate in the study were defined to ensure the suitability and reliability of the sample and they are given below.

## Inclusion criteria:

[1] Students enrolled in the Foundation Course of Phase I MBBS.

[2] Voluntary participation with informed consent

[3] Mandatory presence during the entire session

[4] Capability to comprehend and carry out instructions for self-monitoring of physiological parameters

## Exclusion criteria:

[1] History of diagnosed cardiovascular, respiratory, or psychiatric disorders

[2] Absence from any part of the session or non-submission of feedback forms

## Pre-intervention preparation:

The pre-intervention preparation for the participants before the meditation session, intervention protocol and the post intervention procedure is given in this section.

## Before the session:

Students were oriented to the purpose and protocol of the intervention. They were instructed to observe their breathing, bodily sensations, emotional state and mental activity. A faculty member trained students to measure their pulse rate (via radial artery palpation) and respiratory rate (by counting breaths per minute). A digital stopwatch ensured uniformity in timing. A single stopwatch was run by the teacher for all the students. Each student practiced measurement thrice under supervision to enhance reliability and minimize inter-observer variability.

## Intervention protocol:

Each cohort participated in a single 20-25-minute guided Heartfulness Meditation session, conducted by a certified Heartfulness Meditation trainer.

## The protocol included the following:

[1] Progressive body relaxation

[2] Gentle redirection of attention toward the heart (not visualizing light, but assuming its presence).

[3] Non-judgmental observation of emerging thoughts

[4] Reinforcement to gently return attention to the heart when distracted.

Participants remained seated in silence with closed eyes throughout the session. The setting was optimized for comfort, silence and internalization. Following the meditation session, students were encouraged to sit silently for a few moments and mentally note changes in their thoughts, breathing, emotional state and body awareness.

## Post-intervention procedure:

Immediately after the session, participants were asked to re-record their Pulse and Respiratory Rate using the same technique and asked to fill the structured feedback form to assess the changes in the following parameters.

[1] Calmness and inner stillness

[2] Emotional regulation

[3] Breath depth and ease

[4] Clarity of thought

[5] Body awareness and relaxation

The feedback included optional qualitative responses. The following parameters were recorded.

[1] Pulse Rate (beats per minute) - self-recorded

[2] Respiratory Rate (breaths per minute) - self-recorded

[3] Subjective Experience Feedback - captured through questionnaire.

## Statistical analysis:

The analysis of data was carried out using IBM SPSS Statistics Version 26.0. Primary outcome measures included the change in pulse and respiratory rates before and after the intervention.

The paired t-test was applied to assess statistical significance.

[1] Results were regarded as statistically significant at a p-value of < 0.05.

[2] All continuous variables were presented as mean ± Standard Deviation (SD). Assumptions for parametric testing (normality and homogeneity) were verified before analysis.

## Results:

Table 1 (see PDF) summarizes the pooled effects of Heartfulness Meditation on two key physiological parameters-pulse rate and respiratory rate-measured pre- and post-intervention across all participant cohorts. A statistically significant reduction was observed in both parameters following a single 20-25-minute meditation session. These findings point to a rapid physiological shift toward parasympathetic dominance typically associated with relaxation, calmness and reduced autonomic arousal. Notably, such effects were observed after just a single session of Heartfulness meditation technique, reinforcing its potential utility as a non-pharmacological intervention for stress regulation in high-demand academic settings such as medical schools. Table 2 (see PDF) presents the pooled subjective experiences reported by participants after the guided Heartfulness session. A remarkably high proportion of students (90.4%) reported a sense of relaxation, underscoring the immediate calming effect of the meditation practice. This was accompanied by a slowing of breath in 85.6% of the participants, a reliable indicator of parasympathetic nervous system activation and overall autonomic balance. Nearly three-fourths (75.4%) described their breathing as slow and deep, pointing to increased diaphragmatic breathing, often associated with improved vagal tone. At baseline, 50% of students reported having many thoughts, reflecting a cognitively preoccupied mental state prior to the intervention. 37.6% reported that many thoughts were coming during the session and 66.8 % reported that their mind was clear, calm and focused towards the conclusion of session. Demonstrating a decrease in cognitive chatter by 16.8 %. The progressive quieting of thoughts further supports the calming, inward-focused nature of Heartfulness meditation. This outcome is significant, particularly in a cohort of young students encountering the academic and emotional challenges of a new curriculum. The feeling of lightness in the body (67.2%) and loss of breath awareness (59.2%) suggests early stages of internalized attention, often seen during the onset of meditative absorption or pratyahara. These self-reported experiences align closely with the objective findings (pulse and respiratory rate reductions), validating the possible physiological and psychological synchrony induced by the practice. The present multi-cohort observational study demonstrates that even a single session of Heartfulness Meditation can induce significant reductions in pulse and respiratory rates. These physiological shifts were accompanied by notable improvements in subjective states, including relaxation, enhanced mental clarity and heightened body awareness. Such outcomes suggest that short-term exposure to Heartfulness practice can yield immediate psychophysiological benefits, complementing existing stress-management strategies in medical education.

## Discussion:

The present findings align closely with prior research demonstrating that even brief meditation practices can induce measurable improvements in autonomic and psychological functioning. Like other mindfulness-based interventions, Heartfulness Meditation-which uniquely integrates the element of Yogic Transmission (Pranahuti)-showed significant benefits following a single session. This observation emphasizes the method's practicality, scalability and relevance in environments where time constraints often limit engagement with longer wellness programs. In the context of medical education, these results acquire particular significance. Medical students experience persistent academic pressure, mental fatigue and emotional strain, which collectively compromise cognitive performance and overall well-being. The present studies observed that even a single Heartfulness session was associated with notable reductions in physiological parameters such as pulse rate (PP) and respiratory rate (RR), along with enhanced subjective feelings of calmness, clarity and breathe awareness. Such outcomes indicate that Heartfulness Meditation can serve as a rapid, low-cost and accessible stress management tool for medical trainees. The reproducibility of improvements across multiple cohorts suggests that the observed changes are consistent and reliable. The findings encourage the integration of meditation-based practices into medical training programs, both as preventive and therapeutic measures against burnout and emotional exhaustion. Future research could expand upon these results by exploring the cumulative effects of regular Heartfulness practice on academic performance, resilience and emotional intelligence. With the increasing use of technology in education, Artificial Intelligence (AI) applications could be developed to predict stress levels among students and evaluate the real-time impact of Heartfulness Meditation as a remedial tool. Preliminary neurophysiological evidence indicates that Heartfulness practice may stimulate vagal activity, enhance parasympathetic regulation and broaden conscious awareness- mechanisms that contribute to emotional balance, improved attention and reflective capacity in high-stress academic and clinical environments.

## Limitations:

Despite encouraging results, this study has several limitations. Its cross-sectional design and absence of longitudinal follow-up restrict the ability to evaluate sustained effects over time. The lack of a control group limits causal interpretations and reliance on self-recorded physiological data may introduce measurement bias. Subjective feedback, while valuable for understanding perceived experiences, is inherently prone to response bias. Furthermore, the sample was limited to healthy young adults from a single medical institution, reducing generalizability to other populations. Finally, the intervention consisted of only a single meditation session, providing no insight into potential cumulative or dose-dependent benefits.

## Conclusion:

Single sessions of Heartfulness Meditation produced both statistically and clinically meaningful reductions in physiological markers of stress, along with improvements in perceived calmness and breathe awareness. Thus, we show the potential of Heartfulness Meditation as a rapid, non-pharmacological and scalable method for enhancing psychological well-being in medical trainees. Although preliminary, the results justify larger, controlled and longitudinal studies to evaluate the sustained impact of Heartfulness practice on stress, emotional resilience and cognitive performance among healthcare professionals in training.
